# Correction: Characterization of an exopolysaccharide from probiont *Enterobacter faecalis* MSI12 and its effect on the disruption of *Candida albicans* biofilm

**DOI:** 10.1039/d1ra90123k

**Published:** 2021-06-04

**Authors:** G. Seghal Kiran, S. Priyadharshini, K. Anitha, Elumalai Gnanamani, Joseph Selvin

**Affiliations:** Department of Food Science and Technology, Pondicherry University Puducherry – 605014 India seghalkiran@gmail.com; Department of Chemistry, Stanford University Stanford USA gnanam@stanford.edu; Department of Microbiology, School of Life Sciences, Pondicherry University Puducherry – 605014 India josephselvinss@gmail.com jselvin.mib@pondiuni.edu.in +91-413-2655358 +91-413-2655358

## Abstract

Correction for ‘Characterization of an exopolysaccharide from probiont *Enterobacter faecalis* MSI12 and its effect on the disruption of *Candida albicans* biofilm’ by G. Seghal Kiran *et al.*, *RSC Adv.*, 2015, **5**, 71573–71585, DOI: 10.1039/C5RA10302A.

The authors regret that incorrect versions of Fig. 7 and 8 were included in the original article. The correct versions of Fig. 7 and 8 are presented below with updated captions.

**Fig. 7:**
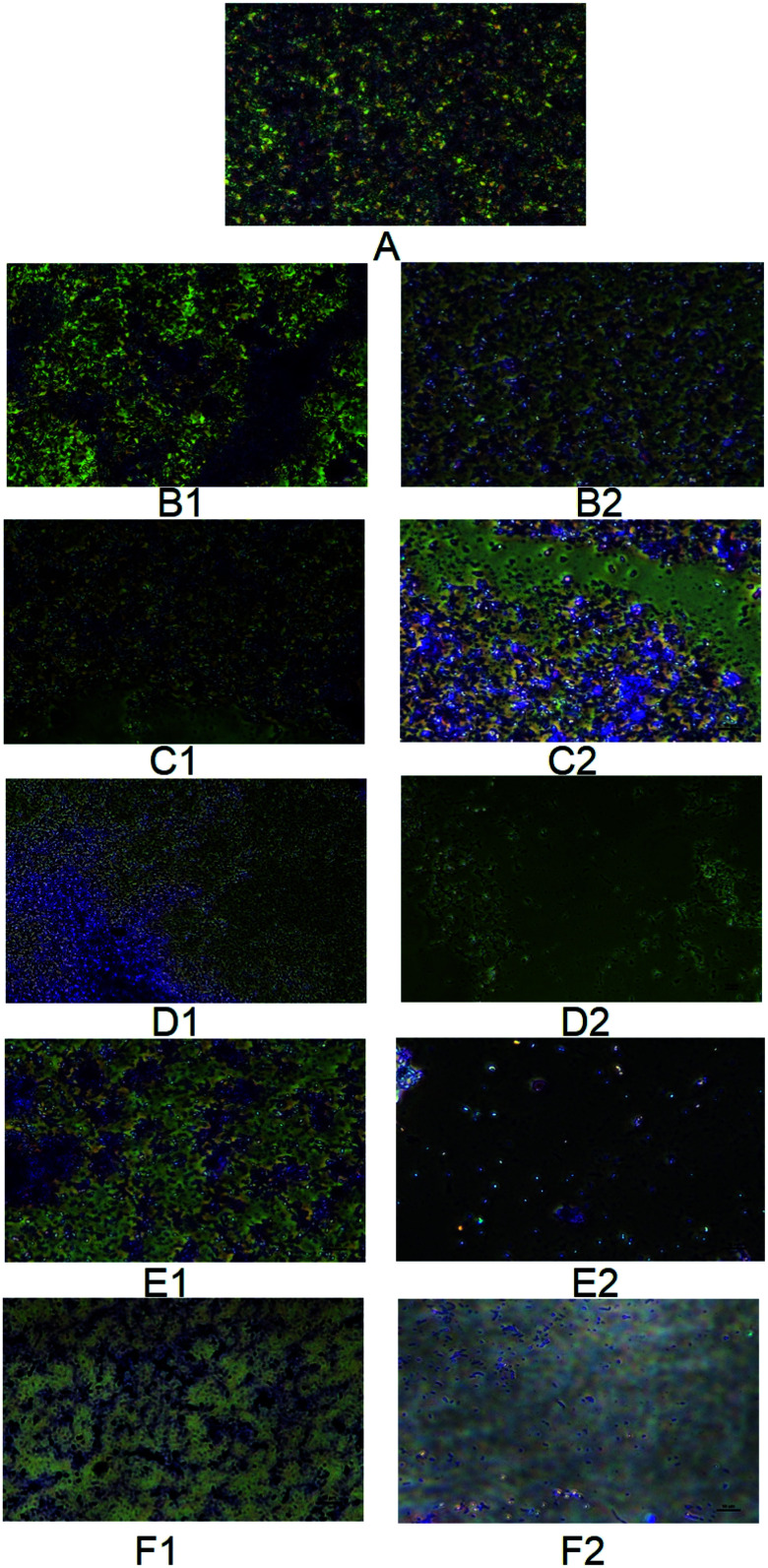
Phase contrast micrographs showing biofilm disruption potential of MSI12-EPS on *C. albicans*. The *C. albicans* biofilm was developed on a cover glass and then it was treated with varying concentrations of EPS and fluconazole ranging from 50–250 μg. The treated cover glass was stained with crystal violet and observed under a phase-contrast microscope (Nikon) at ×40 magnification. A – Control biofilm, B1 – 50 mg fluconazole, B2 – 50 μg MSI12-EPS, C1 – 100 mg fluconazole, C2 – 100 μg MSI12-EPS, D1 – 150 mg fluconazole, D2 – 150 μg MSI12-EPS, E1 – 200 mg fluconazole, E2 – 200 μg MSI12-EPS and F1 – 250 mg fluconazole, F2 – 250 μg MSI12-EPS. The images were recorded using a uniform scale of 10 μm which is shown in the image panels.

**Fig. 8:**
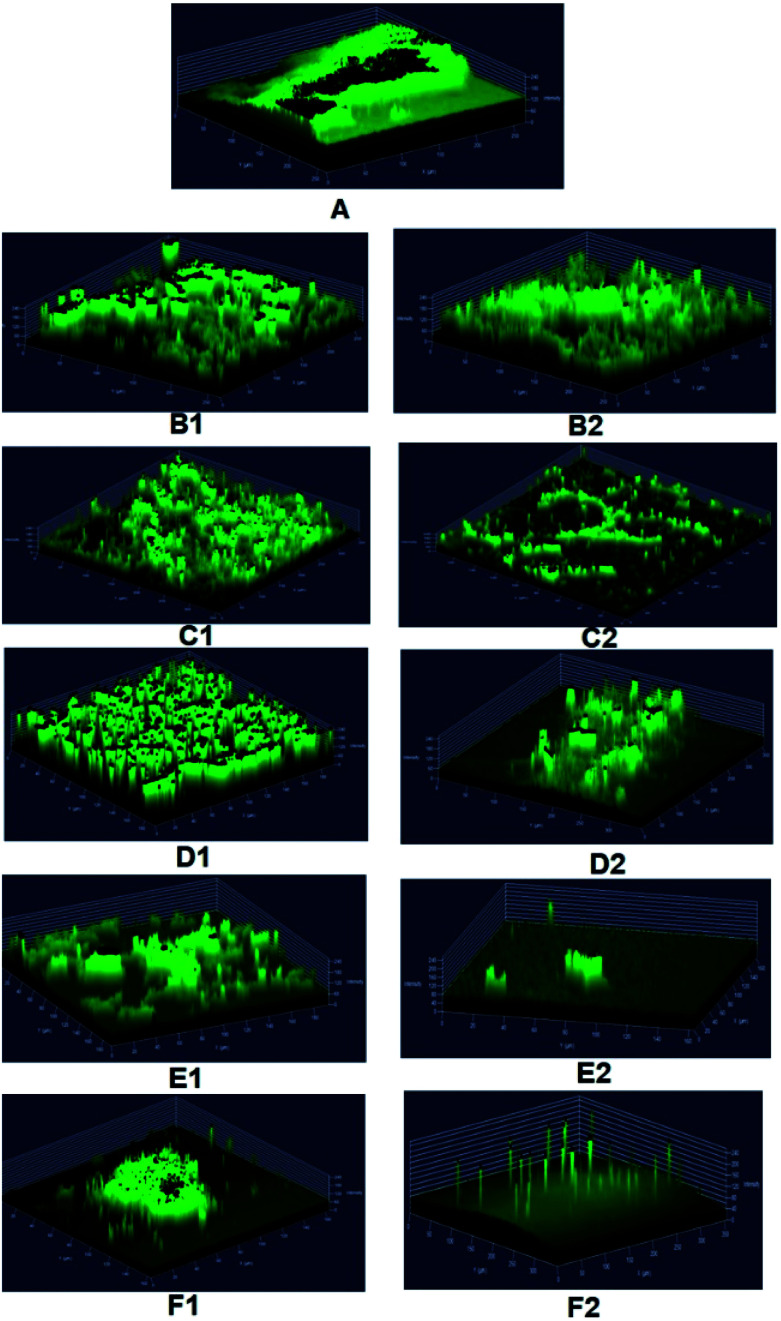
Confocal laser scanning micrographs showing biofilm disruption potential of MSI12-EPS on *C. albicans*. The pre-formed biofilm was treated for 24 h with EPS and fluconazole of varying concentrations ranging from 50–250 μg. Untreated biofilms were used as controls and the biofilm coverage thus formed on glass slides were stained with 0.1% acridine orange and subjected to visualization in a CLSM (LSM 710, Carl Zeiss). A – Control biofilm, B1 – 50 mg fluconazole, B2 – 50 μg MSI12-EPS, C1 – 100 mg fluconazole, C2 – 100 μg MSI12-EPS, D1 – 150 mg fluconazole, D2 – 150 μg MSI12-EPS, E1 – 200 mg fluconazole, E2 – 200 μg MSI12-EPS and F1 – 250 mg fluconazole, F2 – 250 μg MSI12-EPS.

Accordingly, the experimental methods followed in the phase contrast microscopy and large-size images of Fig. 7 have been provided in the ESI. The ESI has been updated online to reflect this change.

The Royal Society of Chemistry apologises for these errors and any consequent inconvenience to authors and readers.

## Supplementary Material

